# Comparative effectiveness and safety of open triple‐branched stent graft technique with stented elephant trunk implantation in treating Stanford type A aortic dissection: A trial sequential meta‐analysis

**DOI:** 10.1111/jocs.16998

**Published:** 2022-11-09

**Authors:** Lelin Bin, Jianbin Fei, Long Zhao, Ruofeng Hong, Wenyu Yang

**Affiliations:** ^1^ Cardiovascular Surgery Department, Hwa Mei Hospital University of Chinese Academy of Sciences Ningbo China

**Keywords:** aortic dissection, meta‐analysis, Stanford classification, triple‐branched stent, type A

## Abstract

**Background:**

The optimal surgical intervention for Stanford type A aortic dissection is controversial. The aim of this trial sequential meta‐analysis was to investigate the comparative effectiveness and safety of open triple‐branched stent graft and stent elephant trunk implantation for total aortic arch reconstruction in Sandford type A aortic dissection.

**Methods:**

PubMed, Embase, Cochrane library, Chinese Biomedical Literature database (CBM), and China National Knowledge Infrastructure (CNKI) were searched for retrieving relevant studies from inception to February 28, 2022. We evaluated 30‐day mortality, procedure‐related time including cardiopulmonary bypass (CPB), aortic cross‐clamp (ACC), and selective cerebral perfusion (SCP), the incidence of postoperative complications including paralysis, cerebral embolism, and acute renal failure, intensive care unit (ICU) time, and medical expenditure. Statistical analysis was performed by RevMan 5.4 and trial sequential analysis (TSA) software.

**Results:**

Six studies involving 260 dissection cases were included eventually. Total aortic arch reconstruction with open triple‐branched stent graft was comparable to the stented elephant trunk implantation in 30‐day mortality, incidence of postoperative complications, ICU time, and medical expenditure, but open triple‐branched stent graft was related to shorter procedure‐related time including CPB (mean difference [MD] = −46.11, 95% confidence interval [CI] = −67.24 to −24.98, *p* < .001), ACC (MD = −42.82, 95% CI = −66.74 to −18.90, *p* < .001), and SCP (MD = −17.88, 95% CI = −33.36 to −2.39, *p* = .02). TSA confirmed robustness of findings.

**Conclusions:**

Our analysis suggested that total aortic arch reconstruction with open triple‐branched stent graft may be an effective and simplified procedure than the stented elephant trunk implantation.

**Abbreviations**
ACC: aortic cross‐clamp
AD: aortic dissection
CBM: Chinese Biomedical Literature database
CI: confidence interval
CNKI: China National Knowledge Infrastructure
CPB: cardiopulmonary bypass
ICU: intensive care unit
MD: mean difference
MeSH: medical subject headings
OR: odds ratio
RevMan: Review Manager
ROBINS‐I: Risk of Bias in Nonrandomized Studies of Interventions
SCP: selective cerebral perfusion
TSA: trial sequential analysis

## 1 INTRODUCTION

Aortic dissection (AD) is the most common acute emergency condition of the aorta, which refers to an arterial disease in which blood entering the media from the vessel lumen causes the aortic lumen presenting a pathological state of true and false lumen due to the intimal tear.^1^ Studies reported 2000 and 3000 new AD cases per year in the United States and in Europe, respectively,^2^, ^3^ with an overall incidence of 1–3/10,000 per year.^4^

The overall outcome of AD is related to the type and extent of dissection. According to the need for surgery, AD was classified into types A and B, which was termed as the Stanford classification system.^5^ Stanford type A AD involves the ascending aorta and may extend into the descending aorta. According to the published studies, Stanford type A AD approximately accounts for 60%–70% of dissection cases, and also represents the most dangerous type of dissection.^1^ Surgical intervention is still the most effective approach for Stanford type A AD.^6^ If untreated with proper surgical interventions, more than 50% patients within 48 h and 75% patients within 2 weeks could die.^7^ Therefore, treatment for Stanford type A AD remains a challenge.

For acute Stanford type A AD, prevention for aortic wall rupture is the main treatment goal. Approximately 90% of acute Stanford type A AD received various surgical treatments, including fenestration, parallel grafting, and branched stent grafting.^8^ However, high incidence of postoperative complications continues to be reported.^9^ It remains controversial on the optimal surgical intervention of Stanford type A AD. Currently, total aortic arch reconstruction with the stented elephant trunk implantation and with open triple‐branched stent graft have been used to treat acute Stanford type A AD in China. However, the comparative effectiveness and safety between the two surgical procedures have not yet been determined. We, therefore, performed this meta‐analysis to systematically investigate the comparative role of open triple‐branched stent graft and the stented elephant trunk implantation in the treatment of acute Stanford type A AD.

## 2 MATERIAL AND METHODS

We performed this study followed the Cochrane handbook and reported results followed the Preferred Reporting Items for Systematic Reviews and Meta‐Analysis statement.^10^ Ethical approval and informed consent were not required because patients and animals were not involved.

### 2.1 Study identification

PubMed, Embase, the Cochrane library, the Chinese Biomedical Literature database (CBM), and China National Knowledge Infrastructure (CNKI) were searched from their inception to February 28, 2021. The search strategy was created using medical subject headings (MeSH) and free words, including “dissecting aneurysm” and “triple‐branched stent graft.” We manually checked the references of eligible studies and reviews to identify additional studies missed from the initial search. Details of the search strategy is summarized in Supporting Information: Table S1. Any discrepancy was solved based on consensus between two reviewers.

### 2.2 Eligibility criteria

Eligible studies were determined according to the following criteria: (a) case–control studies enrolled patients who were definitively diagnosed with Stanford type A AD; (b) patients in the case group received total aortic arch reconstruction with open triple‐branched stent graft but patients in the control group received reconstruction with the stented elephant trunk implantation; and (c) the study reported enough information and outcomes of interest for performing meta‐analysis. Publication status, date, language, and geographical area were not restricted. Studies were excluded if they did not include (a) adequate information, (b) comparison between triple‐branched stent graft and the stented elephant trunk implantation, and (c) outcomes of interest.

### 2.3 Study selection

We first eliminated duplicate studies via EndNote X9 software. The eligibility of identified studies was initially evaluated through screening the titles, abstracts, and keywords. Finally, eligible studies were determined through reviewing the full texts of potentially eligible studies.

### 2.4 Data extraction

The following data were independently extracted from the eligible studies, including basic information, number of cases, gender ratio, mean age of all cases, and outcomes of interest. The primary outcomes included 30‐day mortality and procedure‐related time including cardiopulmonary bypass (CPB) time, aortic cross‐clamp (ACC) time, and circulation arrest or selective cerebral perfusion (SCP) time. Secondary outcomes were incidence of postoperative complications including paraplegia, cerebral embolism, acute renal failure, intensive care unit (ICU) time, and medical expenditure.

### 2.5 Risk of bias assessment

The methodological quality of eligible studies was assessed using the Risk of Bias in Nonrandomized Studies of Interventions (ROBINS‐I) tool.^11^ Three domains including preintervention (bias due to confounding factors and bias in the selection of participants into the study), at intervention (bias in classification of intervention), and prointervention (bias due to deviations from intended interventions, bias due to missing data, bias in the measurement of outcomes, and bias in the selection of the reported result) were used to quantitatively the level of methodological quality. Any discrepancy was resolved based on consensus between the two reviewers.

### 2.6 Statistical analysis

We used odds ratio (OR) with 95% confidence interval (CI) to express the estimates of categorical variables categorical variables, as all data were extracted from case–control studies. In addition, we used mean difference (MD) with 95% CI to express the estimates of continuous variables. We selected the random‐effects model to calculate effect size. We evaluated statistical heterogeneity by utilizing the *χ* ^2^ (Cochran *Q*) test and the *I* ^2^ statistic.^12^ Substantial statistical heterogeneity was supported if *p*< .1 and *I* ^2^ statistic >50%. We performed the sensitivity analysis if the presence of substantial statistical heterogeneity for primary outcomes. Additionally, trial sequential analysis (TSA) was performed to evaluate whether a definitive conclusion could be drawn for the primary outcomes from currently available evidence. The conventional monitoring boundary of ±1.96 was identified, and the TSA monitoring boundary varied by analysis. A definitive conclusion could be drawn if required information size was accessed and/or TSA monitoring boundary was crossed. Moreover, a nonsignificant result would be confirmed if the cumulative *Z*‐curve fell in the futility boundary or the inner wedge of futility.^13^ The models for primary outcomes were assessed based on a significance level of .05 and a statistical power of 80%. A study incidence and control incidence which were calculated from meta‐analysis was set for categorical variables. For continuous variables, MD and variance were calculated from empirical information. Heterogeneity was corrected using model‐based variance. Finally, publication bias was evaluated for primary outcomes using Begg's and Egger's tests. Meta‐analysis was performed using Review Manager (RevMan) (version 5.4, the Nordic Cochrane Centre, the Cochrane Collaboration, Copenhagen, 2014), and influence analysis and publication bias examination were conducted using STATA 14.0 (State Corporation), and the TSA was performed using TSA software version 0.9.5.10 Beta.

## 3 RESULTS

### 3.1 Study selection

After performing preliminary literature search, a total of 184 relevant records were identified. Of which, 61 duplicates were labeled and then removed via EndNote software. After screening titles and abstracts, 10 studies were retained for final evaluation based on full‐text review. Four studies were excluded due to ineligible control (*n* = 1), ineligible patients (*n* = 1), and irrelevant topic (*n* = 2). Therefore, we included six eligible studies^14^, ^15^, ^16^, ^17^, ^18^, ^19^ for this meta‐analysis (Supporting Information: Figure S1).

### 3.2 Study characteristics

Detailed baseline information of six eligible studies is presented in Table 1. All studies^14^, ^15^, ^16^, ^17^, ^18^, ^19^ were performed in China and published between 2009 and 2017. A total of 113 patients were treated by total aortic arch reconstruction with open triple‐branched stent graft and 147 patients received reconstruction with stented elephant trunk implantation. All studies^14^, ^15^, ^16^, ^17^, ^18^, ^19^ reported intraoperative parameters and 30‐day mortality, five studies^14^, ^15^, ^16^, ^17^, ^19^ reported postoperative complications, three studies^14^, ^16^, ^19^ reported ICU time, and two studies^16^, ^17^ reported medical expenditure.

**Table 1:** Baseline information of six eligible studies

References	Country	Number of cases	Gender ratio (male/female)	Mean age, years	Outcomes
Wang et al.^18^	China	25 vs. 25	19/6 vs. 20/5	48 vs. 46	CPB, ACC, SCP, 30‐day mortality,
Shen et al.^17^	China	8 vs. 20	5/3 vs. 12/8	47.1 vs. 50.3	CPB, ACC, SCP, 30‐day mortality, complications, medical expenditure
Li et al.^16^	China	12 vs. 12	9/3 vs. 11/1	53.3 vs. 48.8	CPB, ACC, CCP, 30‐day mortality, ICU time, complications, medical expenditure
Li et al.^15^	China	18 vs. 21	13/5 vs. 16/5	43.5 vs. 45.2	CPB, ACC, SCP, 30‐day mortality, complications
Cheng et al.^14^	China	11 vs. 22	9/2 vs. 18/4	53.8 vs. 46.0	CPB, ACC, SCP, 30‐day mortality, ICU time, complications
Xiong et al.^19^	China	39 vs. 47	33/6 vs. 38/9	50.7 vs. 52.0	CPB, ACC, SCP, 30‐day mortality, ICU time, complications

Abbreviations: ACC, aortic cross‐clamp; CPB, cardiopulmonary bypass; ICU, intensive care unit; NOS, Newcastle–Ottawa Scale; SCP, selective cerebral perfusion.

### 3.3 Risk of bias

Detailed risk of bias is depicted in Supporting Information: Figure S2. All studies^14^, ^15^, ^16^, ^17^, ^18^, ^19^ were labeled with moderate bias due to unclear description on potential cofounding factors and selection of the reported results, but with low bias in the selection of participants, the classification of intervention, and measurement of outcome. One study^18^ was labeled with moderate bias due to deviations from intended interventions and another study^14^ presented moderate bias due to the unclear whether the presence of missing data or not.

### 3.4 30‐day mortality

**Figure 1:**
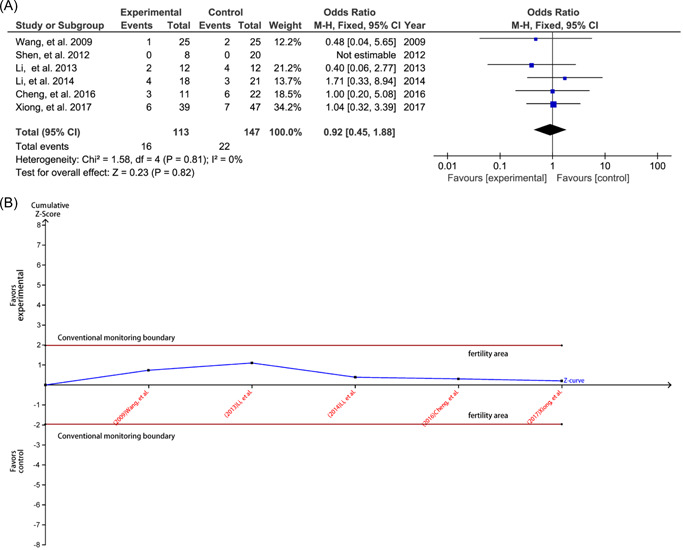
Meta‐analysis and trial sequential analysis for 30‐day mortality

All studies^14^, ^15^, ^16^, ^17^, ^18^, ^19^ reported 30‐day mortality. The pooled result suggested a comparable 30‐day mortality between the two surgical procedures (OR: 0.92; 95% CI: 0.45–1.88; *p* = .82; *I* ^2^ = 0%) (Figure 1A). TSA further demonstrated no difference between the two surgical procedures in terms of 30‐day mortality because the cumulative *Z*‐curve entered the futility area (Figure 1B).

### 3.5 Procedure‐related time

**Figure 2:**
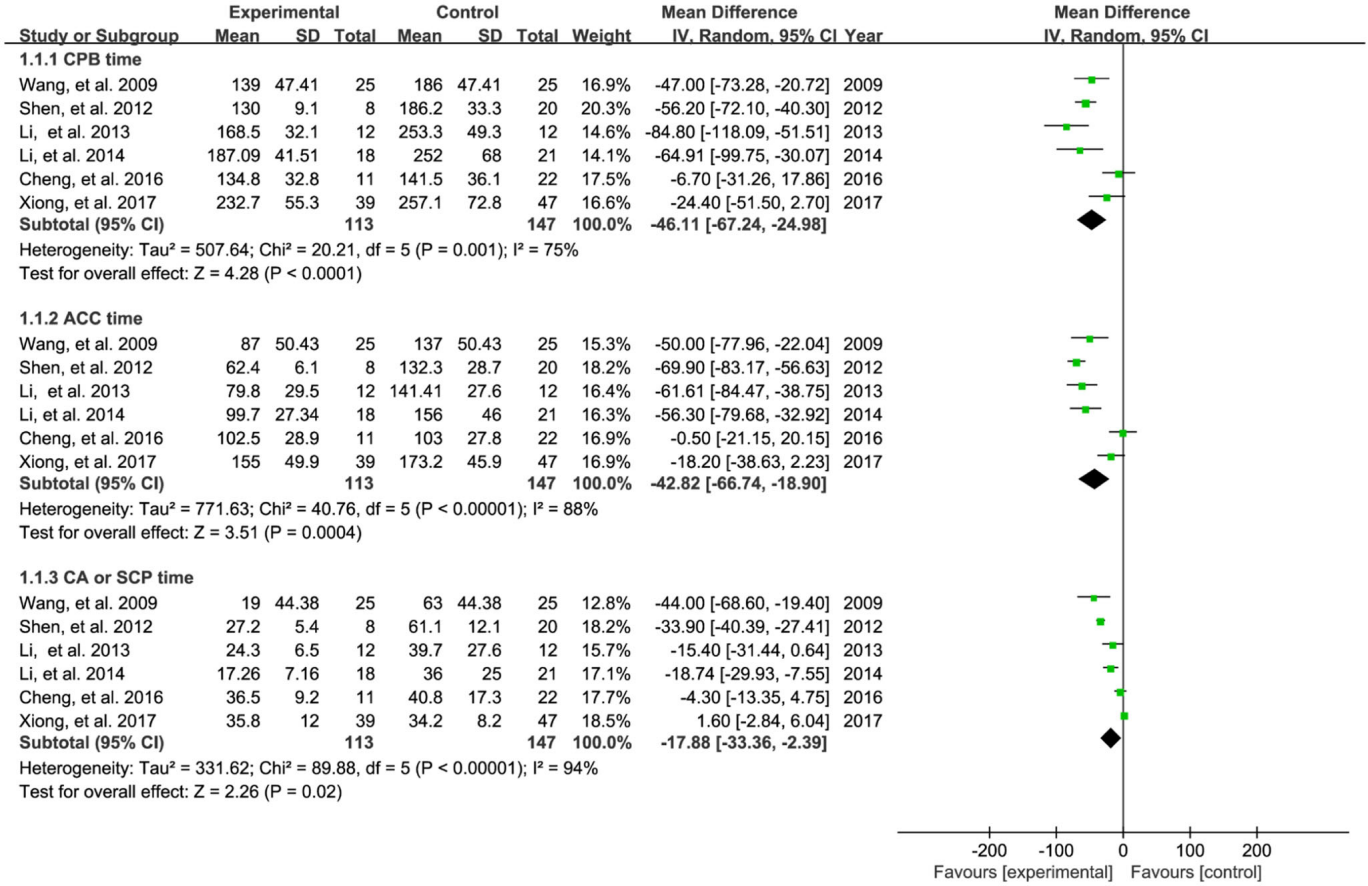
Meta‐analysis for procedure‐related time including CPB, ACC, and SCP. ACC, aortic cross‐clamp; CPB, cardiopulmonary bypass; SCP, selective cerebral perfusion.

All studies^14^, ^15^, ^16^, ^17^, ^18^, ^19^ reported procedure‐related time, including the time of CPB, ACC, and SCP. Pooled results revealed that total aortic arch reconstruction with open triple‐branched stent graft was associated with shorter the time of CPB (MD: −46.11; 95% CI: −67.24 to −24.98; *p* < .001; *I* ^2^ = 75%), ACC (MD: −42.82; 95% CI: −66.74 to −18.90; *p* < .001; *I* ^2^ = 88%), and SCP (MD: −17.88; 95% CI: −33.36 to −2.39; *p* = .02; *I* ^2^ = 94%) (Figure 2). TSA indicated a true positive result for the time of CPB (Supporting Information: Figure S3a) and ACC (Supporting Information: Figure S3b) because the accumulated sample size was adequate and corresponding cumulative *Z*‐curve crossed the sequential monitoring boundary for benefit. However, inconclusive finding was not generated for SCP time because the accumulated sample size was inadequate and cumulative *Z*‐curve did not cross conventional or sequential monitoring boundary (Supporting Information: Figure S3c).

### 3.6 Postoperative complications

**Figure 3:**
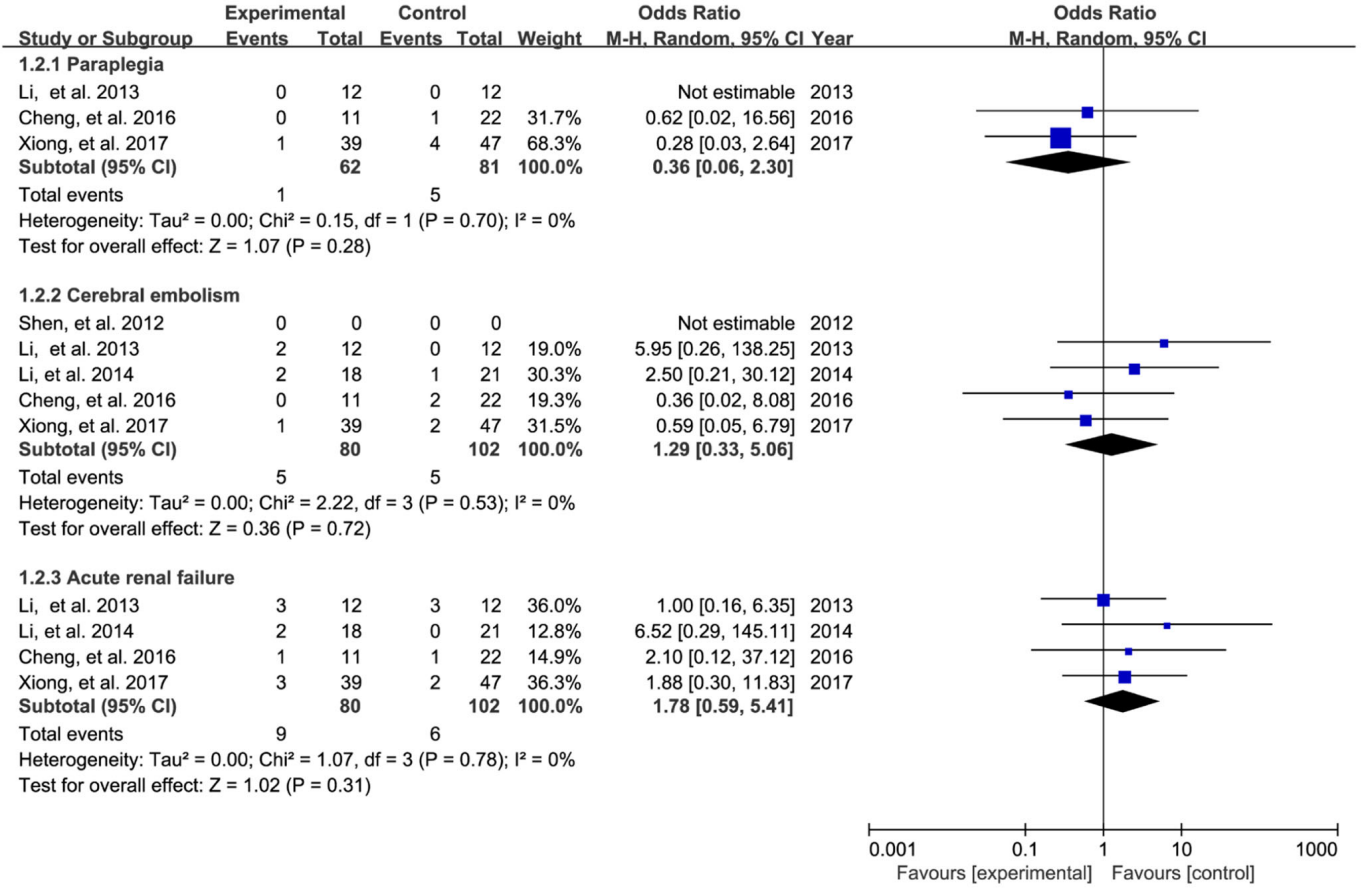
Meta‐analysis for the incidence of postoperative complications

Five studies^14^, ^15^, ^16^, ^17^, ^19^ reported the incidence of postoperative complications. Pooled results revealed no statistical difference between the two surgical procedures in terms of the incidence of paraplegia (OR: 0.36; 95% CI: 0.06–2.30; *p* = .28; *I* ^2^ = 0%), cerebral embolism (OR: 1.29; 95% CI: 0.33–5.06; *p* = .72; *I* ^2^ = 0%), and acute renal failure (OR: 1.78; 95% CI: 0.59–5.41; *p* = .31; *I* ^2^ = 0%) (Figure 3).

### 3.7 ICU time and medical expenditure

Three^14^, ^16^, ^19^ and two^16^, ^17^ studies reported the ICU time and medical expenditure, respectively. Pooled result revealed a comparable ICU time (OR: 2.73; 95% CI: 1.37–5.42; *p* = .004; *I* ^2^ = 84%; Supporting Information: Figure S4a) and medical expenditure (MD: −2.08; 95% CI: −6.66 to 2.49; *p* = .37; *I* ^2^ = 74%; Supporting Information: Figure S4b) between the two surgical procedures.

### 3.8 Sensitivity analysis

In the sensitivity analysis, the pooled point estimates after excluding every study one by one were contained within the 95% CI of the overall pooled results for all outcomes (Supporting Information: Figure S5), indicating that substantial statistical heterogeneity may not impair the reliability of pooled results.

### 3.9 Publication bias

Begg's and Egger's tests revealed no significant asymmetric outline for 30‐day mortality (*z* = 0.73, *p* = .462; *t* = −1.19, *p* = .321), CPB time (*z* = 0.38, *p* = .707; *t* = −0.05, *p* = .960), ACC time (*z* = 0.38, *p* = .707; *t* = 0.90, *p* = .418), and SCP time (*z* = 0.75, *p* = .452; *t* = −1.02, *p* = .365) (Supporting Information: Figure S6), indicating absence of publication bias for all primary outcomes.

## 4 DISCUSSION

The present study is the first one of using meta‐analysis technique to investigate the comparative effectiveness and safety between total aortic arch reconstruction with open triple‐branched stent graft and with the stented elephant trunk implantation for the treatment of Sandford type A AD. Our pooled results based on six case–control studies indicated that, compared with the stented elephant trunk implantation, open triple‐branched stent graft was associated with shorter the procedure‐related time, including the time of CPB, ACC, and SCP, but comparable 30‐day mortality, the incidence of postoperative complications, ICU time, and medical expenditure in the treatment of Stanford type A AD.

Surgical intervention for Stanford type A AD is one of the most challenging and complex clinical problems in adult cardiac surgery although outcomes of surgical procedures for this condition have greatly improved.^20^ Conventional total aortic arch reconstruction procedures are difficult, complex, and invasive.^21^ Therefore, more hybrid techniques have been developed to avoid ACC and hypothermic circulatory arrest.^22^ Although the total arch reconstruction using a tetrafurcate graft with the stented elephant trunk implantation (also named as Sun's procedure) has been indicated as an effective and safe surgical intervention for acute Stanford type A AD,^23^ several shortages limited its application, including difficult distal anastomosis, difficult hemostasis, high risk for phrenic and recurrent laryngeal nerve injury, and long surgery duration.^24^ Therefore, the total arch repair with open triple‐branched stent has been developed to repair the arch in acute type A AD by implanting the triple‐branched stent graft into the proximal descending aorta, arch, and three arch vessels.^25^ Specifically, the surgeon inserted the main stent of the triple‐branched stent graft into the true lumen of the aortic arch and proximal descending aorta through a transverse incision in the ascending aorta, and then positioned each sidearm stent one by one into the aortic branches. Once the main and sidearm stents are properly positioned, the restraint cord is retracted and the main and sidearm stents are deployed. Finally, under the guidance of transesophageal echocardiography, balloon catheter was used to dilate the main and sidearm stents to confirm that they were fully open and free of kinks. The transected distal stump of the ascending aorta was reconstructed with an inner stent‐less Dacron tube and an outer Teflon felt, followed by a continuous anastomosis with a one‐branched Dacron tube.^25^, ^26^, ^27^

Compared to Sun's procedure, by using the open triple‐branched stent graft technique, anastomosis and hemostasis are easier because the distal anastomosis is located at the proximal arch.^28^ Moreover, no additional manipulation was required for the left subclavian artery or descending aorta due to anastomosis of the arch vessels was omitted.^28^ Thus, the technical difficulties of open triple‐branched stent graft are theoretically reduced, which has been supported by our findings because triple‐branched stent graft was associated with shorter procedure‐related time, including CPB, ACC, and SCP. Despite these advantages of triple‐branched stent graft, the following aspects are prerequisites for the successful implementation of this technique and should be paid attention to.^25^ First, an appropriate graft diameter should be designed as this is key to causing rapid clot formation and false lumen retraction, as well as preventing new intimal damage due to the continued compression of an oversized stent graft on the dissected and fragile intimal wall. Second, the surgeon must ensure that the distance between two adjacent sidearm stents is equal to the distance between two corresponding arch vessels, which will keep the sidearm stent grafts from twisting or kinking after deployment.

In this meta‐analysis, we used several statistical methods to further enhance the precision and robustness of pooled results. We calculated all estimates based on a random‐effects model irrespective of the level of statistical heterogeneity because variations between studies could not be eliminated in real settings. Meanwhile, we performed sensitivity analysis (also named as influence analysis) to examine whether variations between studies might impair the reliability and robustness of pooled results via leave one out method. Result of sensitivity analysis indicated that our pooled results were robust and reliable. For primary outcomes, we performed TSA to determine whether a definitive conclusion could be drawn from the available evidence. It's noted that further studies were not required for the confirmation of 30‐day mortality CPB time, and ACC time, however, inconclusive finding could not be generated for SCP time. Certainly, we also used the risk of bias tool for nonrandomized studies to quantitatively the methodological quality, which benefited for clinical decision‐making based on our findings.

We must acknowledge that there are several limitations to this meta‐analysis. The main limitation was the limited numbers of eligible studies and cases. We only included six studies for data analysis; therefore, conclusive findings could not be generated. Furthermore, the result of TSA indicated a need for more studies in terms of SCP time. For comparison between two interventional procedures, randomized controlled trial was the golden standard. However, only case‐control studies investigated the difference between two procedures currently. Therefore, we could not eliminate the negative impact of some potential confounding factors on our findings. Although we searched five international and domestic electronic databases, we did not identify studies performed in other countries except for China to meet our selection criteria. Thus, our conclusions might not applicable to other cultural settings. Certainly, we must also acknowledge that our formal protocol was not registered publicly although we designed the present meta‐analysis strictly followed the recommendations made by the Cochrane handbook.

## 5 CONCLUSION

The total AD with open triple‐branched stent graft is an effective and simplified procedure for treating Stanford type A AD because it has comparable 30‐day mortality, the incidence of postoperative complications, ICU time, and medical expenditure but shorter procedure‐related time to the stented elephant trunk implantation. Future studies with random design should be performed to demonstrate the effects and safety of open triple‐branched stent graft in the treatment of Stanford type A AD.

## AUTHOR CONTRIBUTIONS

Wenyu Yang, Jianbin Fei, Long Zhao, and Lelin Bin carried out the studies, participated in collecting data, and drafted the manuscript. Ruofeng Hong performed the statistical analysis and participated in its design. Wenyu Yang, Jianbin Fei, Long Zhao, and Lelin Bin participated in the acquisition, analysis, or interpretation of data and draft the manuscript. All authors read and approved the final manuscript.

## CONFLICT OF INTEREST

The authors declare no conflict of interest.

## Supporting information

Supplementary information.

Supplementary information.
