# Supplementary material for: Comparative effectiveness and safety of open triple‐branched stent graft technique with stented elephant trunk implantation in treating Stanford type A aortic dissection: A trial sequential meta‐analysis
Source: J Card Surg. 2022 Nov 9;37(12):5210–7. doi: 10.1111/jocs.16998 (PMC10100206; doi:10.1111/jocs.16998)
Supplement: Supplementary file 1 — Supplementary information. [file JOCS-37-5210-s001.docx]

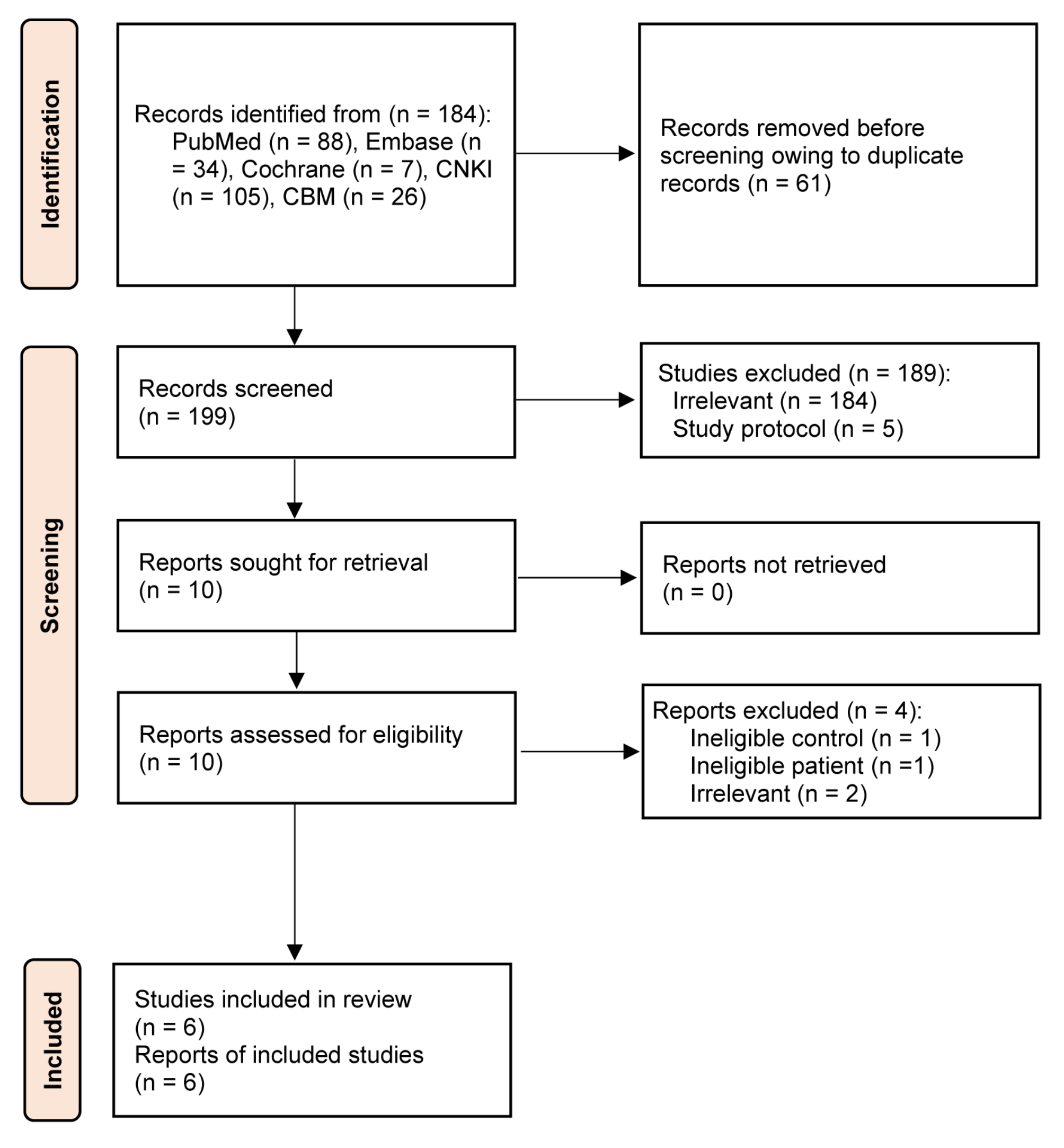


**Figure S1.** PRISAM flow chart of study selection.


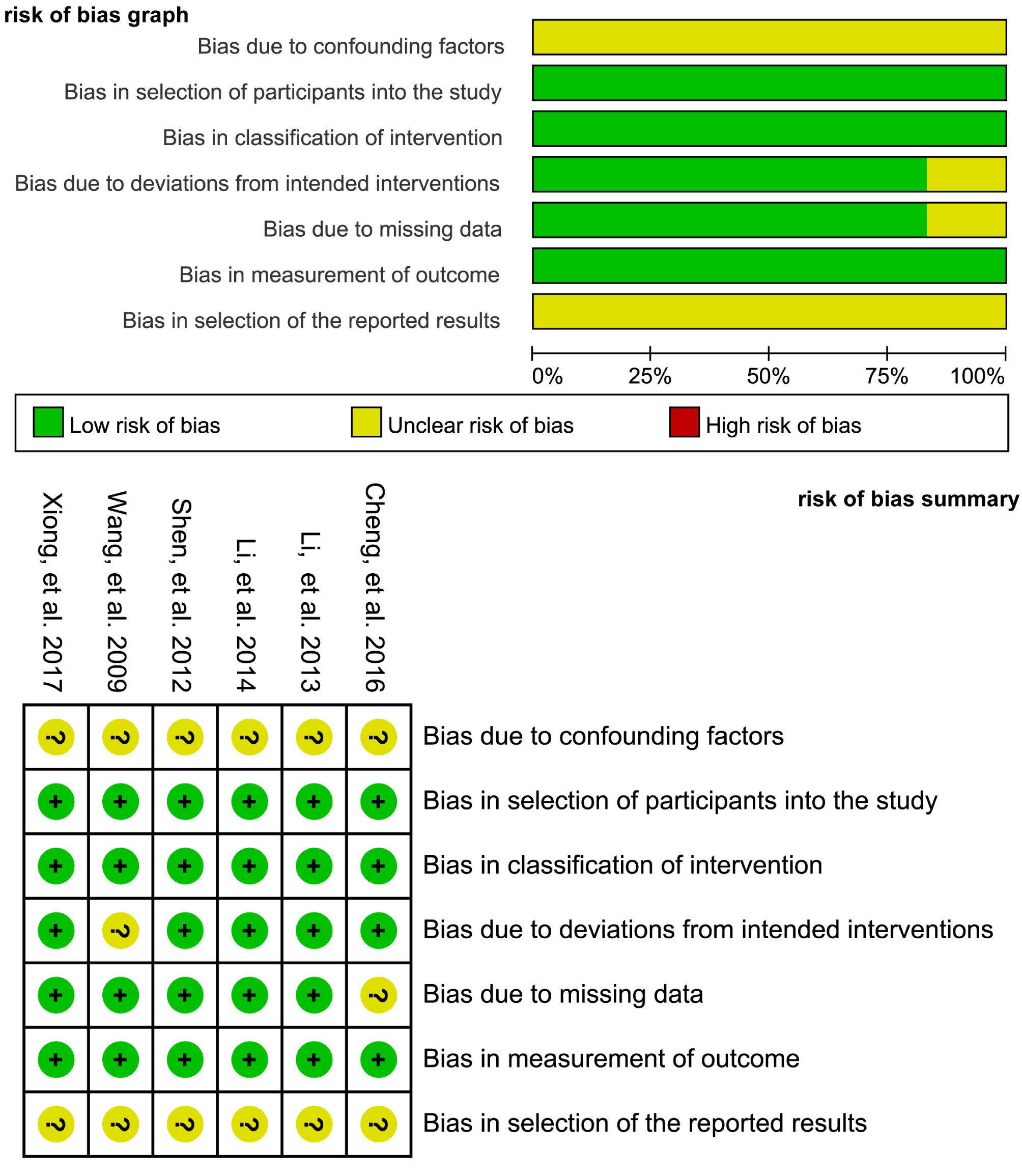


**Figure S2.** Risk of bias assessment for eligible studies.


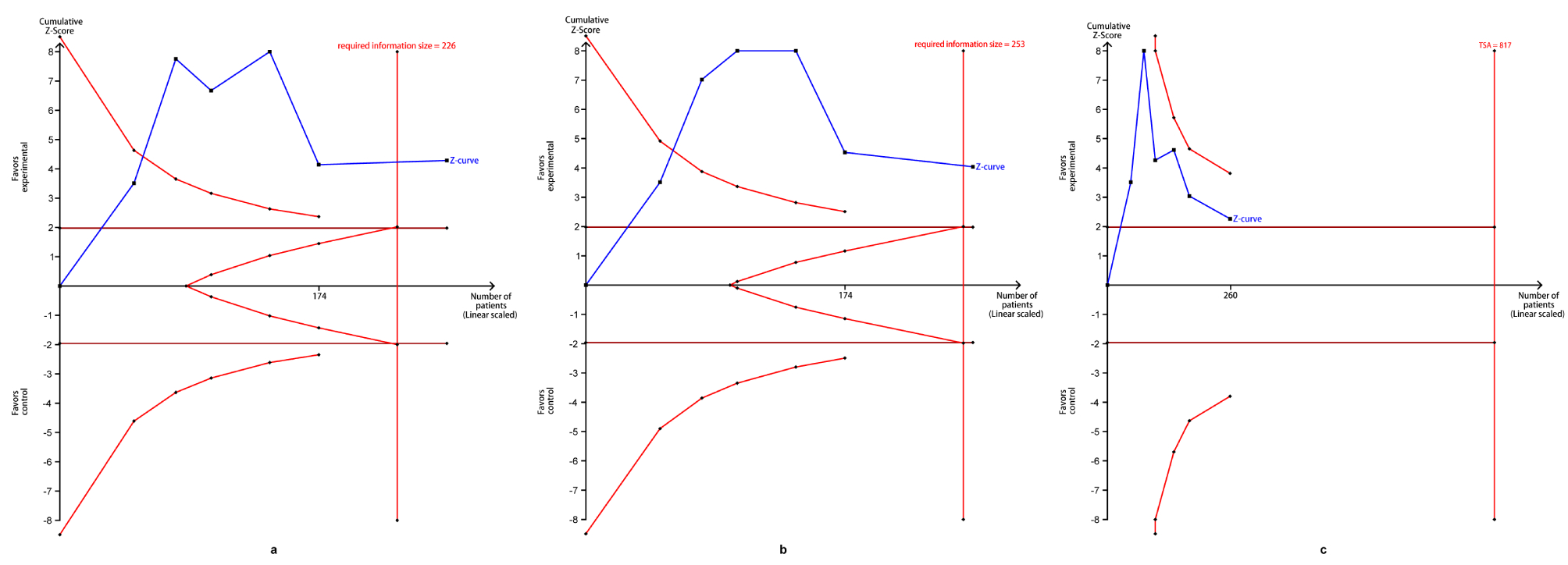


**Figure S3.** Trial sequential analysis for procedure-related time including (a) cardiopulmonary bypass time, (b) aortic cross-clamp time, and (c) circulatory arrest or selective cerebral perfusion time.


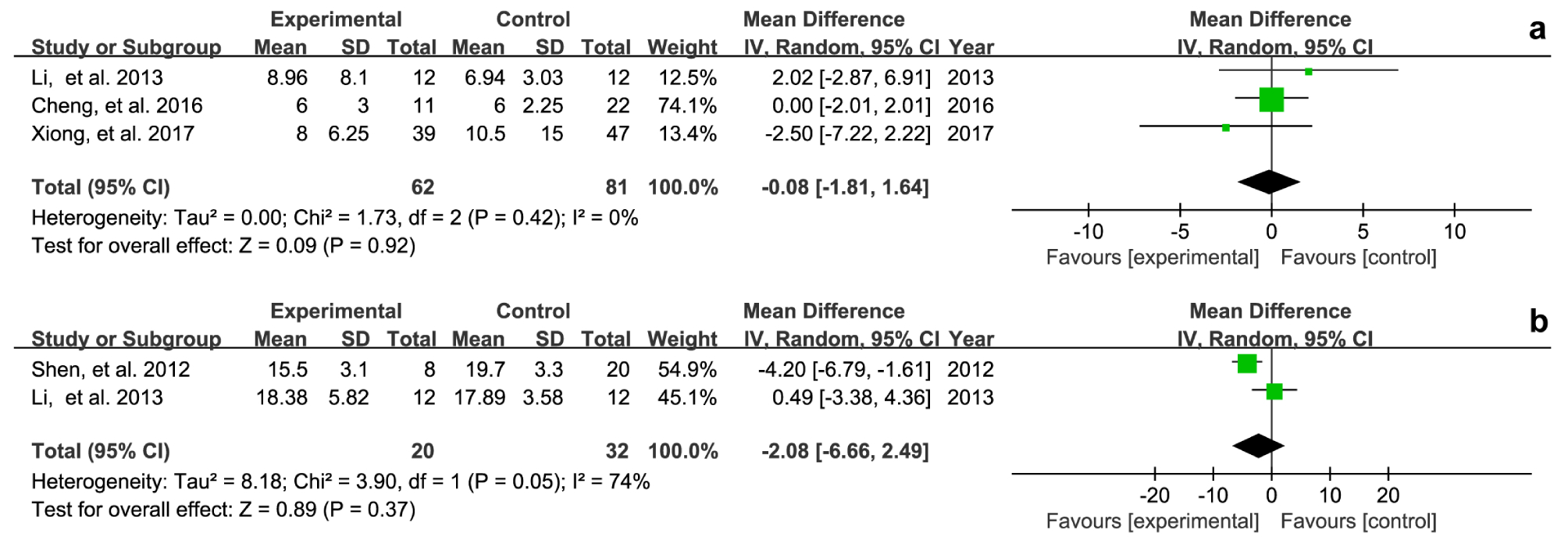


**Figure S4.** Meta-analysis for ICU time (a) and medical expenditure (b). ICU, intensive care unit.


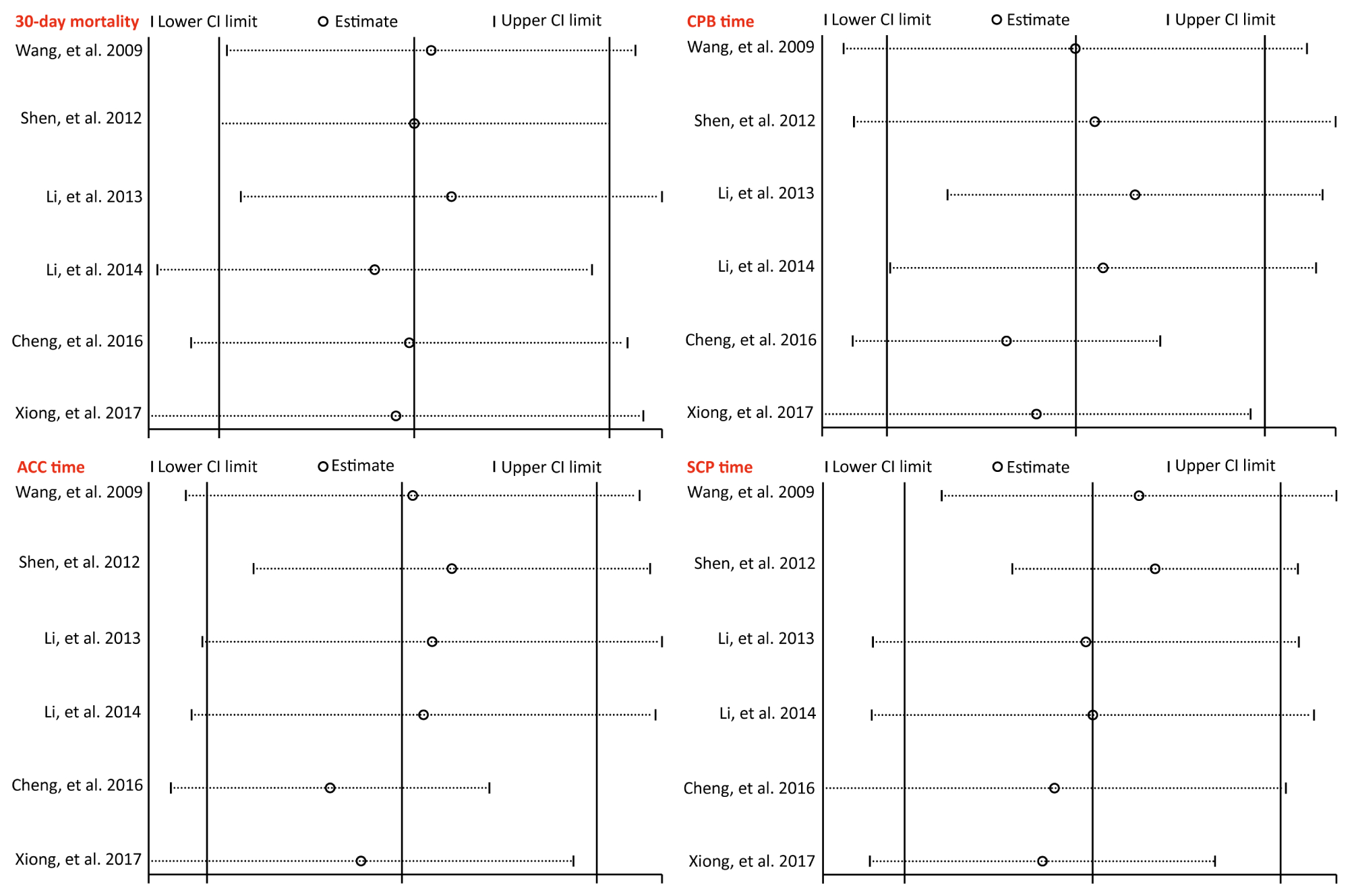


**Figure S5.** Sensitivity analysis for primary outcomes. CPB, Cardiopulmonary bypass; ACC, Aortic cross-clamp; SCP, selective cerebral perfusion; CI, confidence interval.


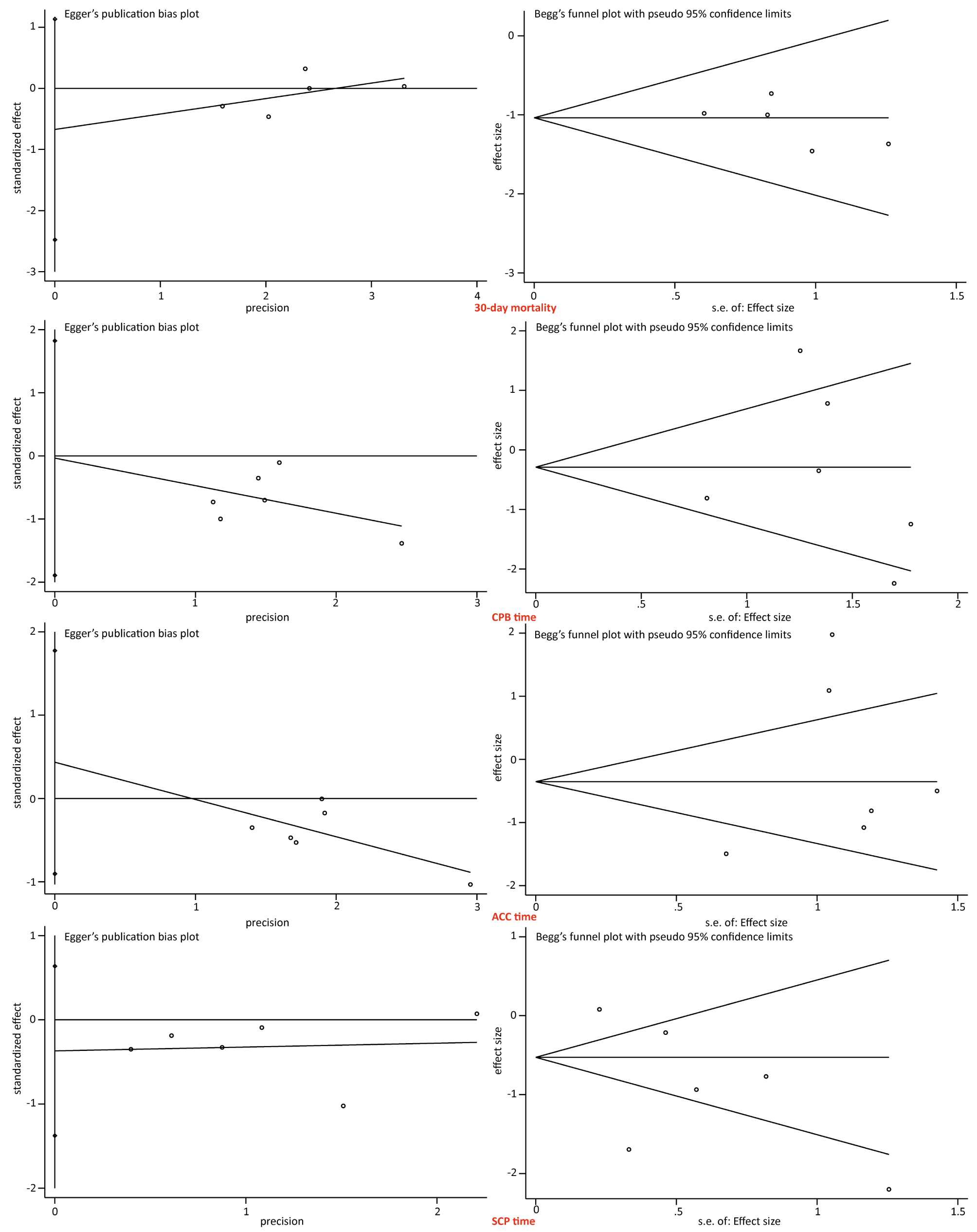


**Figure S6.** Publication bias examination for primary outcomes. CPB, Cardiopulmonary bypass; ACC, Aortic cross-clamp; SCP, selective cerebral perfusion.
