# Supplementary material for: Comparative effectiveness and safety of open triple‐branched stent graft technique with stented elephant trunk implantation in treating Stanford type A aortic dissection: A trial sequential meta‐analysis
Source: J Card Surg. 2022 Nov 9;37(12):5210–7. doi: 10.1111/jocs.16998 (PMC10100206; doi:10.1111/jocs.16998)
Supplement: Supplementary file 2 — Supplementary information. [file JOCS-37-5210-s002.docx]

**Table S1** Search strategy for English target databases.

**Search strategy of PubMed**

.......................................................

Search number Query Results

5 (("Aneurysm, Dissecting"[Mesh]) OR (((((Dissecting Aneurysm[Title/Abstract]) OR (Dissecting Aneurysms[Title/Abstract])) OR (Blood Vessel Dissection[Title/Abstract])) OR (Aortic Dissection[Title/Abstract])) OR (Aortic Dissections[Title/Abstract]))) AND ((((triple-branched stent graft[Title/Abstract]) OR (triple-branched aortic arch covered stent graft[Title/Abstract])) OR (three branches stent graft[Title/Abstract])) OR (three branches aortic arch covered stent graft[Title/Abstract])) 88

4 (((triple-branched stent graft[Title/Abstract]) OR (triple-branched aortic arch covered stent graft[Title/Abstract])) OR (three branches stent graft[Title/Abstract])) OR (three branches aortic arch covered stent graft[Title/Abstract]) 282

3 ("Aneurysm, Dissecting"[Mesh]) OR (((((Dissecting Aneurysm[Title/Abstract]) OR (Dissecting Aneurysms[Title/Abstract])) OR (Blood Vessel Dissection[Title/Abstract])) OR (Aortic Dissection[Title/Abstract])) OR (Aortic Dissections[Title/Abstract])) 29,142

2 ((((Dissecting Aneurysm[Title/Abstract]) OR (Dissecting Aneurysms[Title/Abstract])) OR (Blood Vessel Dissection[Title/Abstract])) OR (Aortic Dissection[Title/Abstract])) OR (Aortic Dissections[Title/Abstract]) 19,067

1 "Aneurysm, Dissecting"[Mesh] 22,438

......................................................

**Search strategy of Embase**

.......................................................

No. Query Results Results Date

#5. #3 AND #4 34

#4. 'triple-branched stent graft':ti,ab,kw OR 39

'triple-branched aortic arch covered stent

graft':ti,ab,kw OR 'three branches stent

graft':ti,ab,kw OR 'three branches aortic arch

covered stent graft':ti,ab,kw

#3. #1 OR #2 28,362

#2. 'dissecting aneurysm'/exp 8,360

#1. 'dissecting aneurysm':ti,ab,kw OR 'dissecting 25,131

aneurysms':ti,ab,kw OR 'blood vessel

dissection':ti,ab,kw OR 'aortic

dissection':ti,ab,kw OR 'aortic

dissections':ti,ab,kw

.......................................................

**Search strategy of Cochrane library**

.......................................................

ID Search Hits

#1 (Dissecting Aneurysm):ti,ab,kw OR (Dissecting Aneurysms):ti,ab,kw OR (Blood Vessel Dissection):ti,ab,kw OR (Aortic Dissection):ti,ab,kw OR (Aortic Dissections):ti,ab,kw 869

#2 MeSH descriptor: [Aneurysm, Dissecting] explode all trees 129

#3 #1 or #2 876

#4 (triple-branched stent graft):ti,ab,kw OR (triple-branched aortic arch covered stent graft):ti,ab,kw OR (three branches stent graft):ti,ab,kw OR (three branches aortic arch covered stent graft):ti,ab,kw 11

#5 #3 and #4 7

.......................................................
